# Trained volunteers to support chronically ill, multimorbid elderly between hospital and domesticity – a systematic review of one-on-one-intervention types, effects, and underlying training concepts

**DOI:** 10.1186/s12877-019-1130-2

**Published:** 2019-05-02

**Authors:** Anne Goehner, Cornelia Kricheldorff, Eva Maria Bitzer

**Affiliations:** 1 Center for Geriatric Medicine and Gerontology Freiburg, Medical Center, Faculty of Medicine, University of Freiburg, Lehener Str. 88, 79106 Freiburg, Germany; 2 Catholic University of Applied Sciences Freiburg, Karlstr. 63, 79104 Freiburg, Germany; 3 University of Education Freiburg, Public Health & Health Education, Kunzenweg 21, 79117 Freiburg, Germany

**Keywords:** Volunteers, Aged, Chronic disease, Hospitals, Aftercare, Lay helper, Support, Accompaniment, Training, Frailty

## Abstract

**Background:**

New approaches are needed to address the challenges of demographic change, staff shortages, and societal change in the care of the elderly. While volunteering has barely been established as a pillar of the welfare state in several countries, legislators and nonprofit or community-based organizations in some countries favor the increased integration of volunteers, as they can rely on many dedicated people. When caring for the multimorbid elderly, the transition from hospital to domesticity involves certain risks. Currently, no systematic knowledge exists on whether and how elderly benefit from volunteer support after a hospital stay. Objectives of this systematic review were to (1) identify evaluated approaches with trained volunteers supporting chronically ill, multimorbid elderly one-on-one at the interface between hospital and domesticity; (2) investigate the patient-related effectiveness of the approaches; (3) present the characteristics of the supporting volunteers; and (4) present the underlying teaching and training concepts for the volunteers.

**Methods:**

A systematic search of the following online databases was conducted in April 2017: the Cochrane Library, Medline (PubMed), CINAHL, and PsycINFO (Ebscohost). We included (cluster/quasi-) randomized controlled trials, controlled clinical trials and single-group pre-post design. An institutional search was conducted on eight national institutions from research and practice in Germany. Screening was conducted by one researcher, risk of bias was assessed. Study authors were contacted for study and training details.

**Results:**

We identified a total of twelve studies, eight of which evaluated treatment following hospital stay: psychosocial-coordinative support (*n* = 2), physical-cognitive activation (*n* = 4), and assistance with medication intake (*n* = 2). We saw short-term effects with small and medium effect sizes. Most volunteers were women aged between 45 and 61 years. Their training lasted 12–26 h and took place prior to first patient contact. During the intervention, volunteers could rely on permanent supporting structures.

**Conclusions:**

Few studies exist that have evaluated one-on-one-volunteer support following hospitalization, and the effects are inconsistent. As such, further, well-designed studies are needed. The suitability and transferability of the interventions in country-specific settings should be examined in feasibility studies. Furthermore, an international discussion on the appropriate theoretical backgrounds of volunteer training is needed.

**Electronic supplementary material:**

The online version of this article (10.1186/s12877-019-1130-2) contains supplementary material, which is available to authorized users.

## Background

In 2017, 13% of the world’s population was older than 60 years. In Europe and the United States, the proportion is highest at 25 and 22%, respectively [1]. By 2050, the proportion of over-60s in all regions of the world, except Africa, will be nearly a quarter or more of the population. The proportion of over-80s will triple from 137 million to 425 million over the same period [1]. The likelihood of chronic diseases, multimorbidity, and their associated risks, as well as care needs increase with age [2]. Therefore, health and social systems must find a way to address great challenges in the coming years: The number of people needing care is increasing, while the resources available for care provision are decreasing – both in terms of the number of caring families, as well as the number of professionals available to assist [3]. The interface between inpatient and outpatient care of the elderly with multimorbid or chronic conditions requires special attention, as risk factors and a lack of supportive resources can lead to a discontinuation of care and, as a consequence, to hospital readmissions [2, 4–8]. Further, those who are discharged back to their own homes – especially those who live alone – will require help with various things, including carrying out their activities of daily living. If a corresponding support network is missing and an individual’s mobility is limited, statutory or voluntary support becomes necessary [9]. Nurse-based approaches include e.g. discharge planning, written discharge instructions, patient education, in-hospital or home visits, and follow-up phone calls [10].

At the same time, against the backdrop of social and demographic developments, care is being increasingly viewed as a task for society as a whole, as exemplified by caring communities. Further, in Germany, for example, legislators and nonprofit or community-based organizations in the field favor the increased integration of volunteers [11–14]. While volunteering has barely been established in some countries as a pillar of the welfare state, individual countries such as Germany already rely on large numbers of dedicated people. An analysis of general volunteering rates based on cumulative data from the European Social Survey (ESS) revealed that while the volunteer rates in countries such as the United Kingdom, Poland, and Portugal remained relatively low (at 4–9%) in organizations and associations, the total commitment rate in Germany between 2002 and 2012 increased from 16.7 to 31.4%, and in Sweden, the rate increased from 24.5 to 34.6% [15].

The added value of volunteers in the support of the elderly has been reflected in recent international studies. In hospital and nursing homes, volunteers have already been providing assistance to mobilize patients [16], prevent delirium [17–20], assist in mealtimes [21–27], and offer everyday help [28], orientation [29], psychosocial support [30], cognitive stimulation [31], or provide accompaniment for persons with dementia [32]. In the community, volunteers offer (educational) courses that are mostly group-based with a focus on self-management or healthy behaviors. Target groups of these initiatives currently include those with several chronic conditions [33–40], diabetes [41–43], heart failure [44], hypertension [45, 46], stroke [47, 48], myocardial infarction [49], cancer [50–52], asthma [53], limb loss [54], or chronic obstructive disease [55]. Further, volunteers offer physical activation [56–61] or fall prevention [62–66] services in the community, that are predominantly group based, too. In addition, there are community-based approaches in which volunteers promote cardiovascular health awareness [67–72], encourage individuals to increase cancer-screening rates [73–76], or support persons with dementia and their relatives at home [77, 78]. Also, in palliative care, volunteers visit or offer activities to seriously ill and dying people [79–82].

While one-on-one-approaches to a better transition of care exist and volunteers have already taken on numerous (mostly group-based) tasks, there is currently a lack of systematic knowledge on whether, and how, chronically ill, multimorbid elderly can benefit from one-on-one support by trained volunteers at the interface between hospital and domesticity.

In recent years, and as part of current social and demographic developments, politicians and nonprofit or community-based organizations have become increasingly convinced that volunteers should be qualified to carry out their work [3, 14]. As part of these developments, some publications have addressed training structures and contents, and their evaluation (e.g. [83–94]). However, information on how to train volunteers in care has not yet been published in sufficient detail [93].

The primary aims of this systematic review were to (1) identify evaluated approaches that employ trained volunteers who provide one-on-one support to chronically ill, multimorbid elderly at the interface between hospital and domesticity (these studies include PPs, CTs, and RCTs), and (2) investigate the patient-related effectiveness of these approaches. Our secondary aims were to (3) present the characteristics of the volunteers in these evaluations, and (4) present the underlying teaching and training concepts that were used to enhance the skills of volunteers.

## Methods

This systematic review followed the guidance from the PRISMA Statement [95]. For the complete PRISMA checklist, see Additional file 1. The entire project was registered in the health services research database (http://www.versorgungsforschung-deutschland.de) under registration number VfD_17_003870.

### Data sources and search criteria

A systematic search of the online Cochrane Library, Medline (PubMed), CINAHL, and PsycINFO (Ebscohost) databases was conducted in April 2017. The search strategy was developed based on the PI(C)OS model (which includes Participants, Intervention, Outcomes, and Study design), pilot searches and published search strategies of previous reviews [40, 94, 96–99]. Specific strategies were developed for each database using a combination of Medical Subject Headings (MeSH) –Terms (where applicable), subject headings, and text terms. Please see Additional file 2 for additional detail.

If our search identified relevant study protocols, we verified whether the results were published by April 2017. If our search identified reviews, we checked whether the included studies met our inclusion criteria. Assuming the search would be unsatisfactory [14], it was supplemented by a search that focused on institutions offering voluntary engagement. The search was conducted on German institutions focused on research and practice, including the Federal Ministry of Health (BMG); the Federal Ministry for Family Affairs, Senior Citizens, Women and Youth (BMFSFJ); the National Association of Statutory Health Insurance Funds (GKV); the Federal Ministry of Education and Research (BMBF); the Social Welfare Association VdK; the Federal Association of Non-statutory Welfare (BAGFW); the German National Association of Senior Citizens’ Organisations (BAGSO); and the National Network for Civil Society (BBE). We searched their online presence for further concepts, not published otherwise. We searched nationally for two reasons. First, due to the limited data available in the field, a supplementary within-organizations search made sense. Second, it was necessary to examine whether an institutional search generated additional results. Our group did not have the expertise required to locate international institutions.

We reviewed the online presence of those national institutions that were identified during the institutional search to determine whether they had additional curricula that subsumed several training concepts or were developed on a scientific basis (e.g., by survey). To obtain further details about volunteer training, we contacted the authors of the included studies. Per our pre-established checklist, we asked for details about the volunteers’ initial training, as well as for the theoretical background and additional support provided to the volunteers following the initial training. Our checklist followed the Template for Intervention Description and Replication (TIDieR) checklist [100]. The curricula were tabulated and analyzed by qualitative content analysis [101]. The focus of this analysis was to examine the training topics, goals, contents, duration, didactic methods, tools, and number plus qualification of the responsible trainer(s).

### Inclusion criteria

Following the German Voluntary Survey [102], “volunteer” was defined as follows: “The commitment is 1. voluntary, 2. free of charge (maximum reimbursement of expenses or overhead lump sum), 3. publicly and 4. jointly exercised with others”.

We included studies if they met our definition of “volunteer”, as well as the following criteria:

- *Population:* Participants who had a mean age at baseline of ≥65 years, with at least one chronic primary diagnosis. (This review focused on participants with chronic, multimorbid conditions). Pilot searches that included the terms “chronic”, “multimorbid”, or “comorbid” lacked findings, as participants of studies exploring geriatric and psychogeriatric interventions were often defined by their primary diagnosis. In addition, the studies suggested that a high proportion (62–80%) of elderly (those ≥65 years) had multimorbid conditions [103]. Therefore, the search strategy included elderly subjects with at least one chronic primary diagnosis.
- *Intervention:* Studies were included if they evaluated transitional care support delivered one-on-one at home, as offered by volunteers who participated in training. Studies were excluded if:
  - they used only ‘expert patients’ as volunteers,
  - the volunteers were only deployed to screen study participants,
  - the intervention was attached to a formalized voluntary service (i.e., a contractually fixed commitment period with a high number of working hours, as is the case for the “Voluntary Social Year” in Germany, which is usually characterized by 39 working hours per week),
  - the intervention was attached to an exchange platform, and
  - the intervention addressed palliative patients.
- *Setting:* Participants home after a hospital stay. Based on the results of previous reviews from related fields, a limited number of studies was to be expected. To face this, additional studies featuring domestic settings were included. To ensure high transferability, interventions were only presented in detail if they were implemented at least once as treatment following a hospital stay.
- *Outcome:* All patient-related outcomes were assessed.
- *Study Design:* (Cluster/quasi-) randomized controlled trials (RCTs), controlled clinical trials (CCTs), and single-group pre–post design (PP) studies were included. We excluded studies with missing pre-survey (only post-survey) data, reports without study results, studies without patient-related data, and reviews without inclusion-appropriate studies.
- *Other criteria:* Studies were included if they were published within the last 15 years (2002–2017), and were presented in either English or German.

### Study selection, data extraction, and synthesis

Based on the inclusion criteria, the first author screened all titles and abstracts for their eligibility. A 20% random full-text sample was independently screened by the first and third authors, and the agreement rate was determined. The first author performed the full-text assessment; in the event of ambiguity, the third author was consulted.

The first and second authors developed and discussed standardized data-extraction sheets for the study characteristics and outcomes. The first author then extracted the study characteristics, including information on study design, setting, sample, characteristics of the intervention and control groups, characteristics of the volunteers, training/supervision for the volunteers, as well as methods, time points, and instruments for the outcome assessments. Data extraction of outcomes, undertaken by the first author, included information on sample size, baseline and follow-up values, the direction of effects, within-group and between-group median group differences, effect size, Cohen’s d, and significance. If these elements were lacking and the data were sufficient, we calculated the mean difference and effect size (Cohen’s d). The following Cohen’s d values were used to indicate effect sizes: 0.2 was interpreted as a small effect, 0.5 was a medium effect, and 0.8 was a large effect [104]. Mean differences and effect sizes disfavoring the intervention were labelled with negative signs. For RCTs and CCTs, between-group significance was presented; for PPs, within-group significance was presented.

Study characteristics and outcomes were descriptively summarized. If the data level was sufficient, a meta-analysis was carried out.

### Quality assessment

The risk of bias at the study level was assessed using the Cochrane Risk of Bias Tool for randomized trials and the Acrobat-NRSI for non-randomized trials [105, 106]. If information on study quality was lacking, we contacted the authors of those studies to request further information.

## Results

**Fig. 1:**
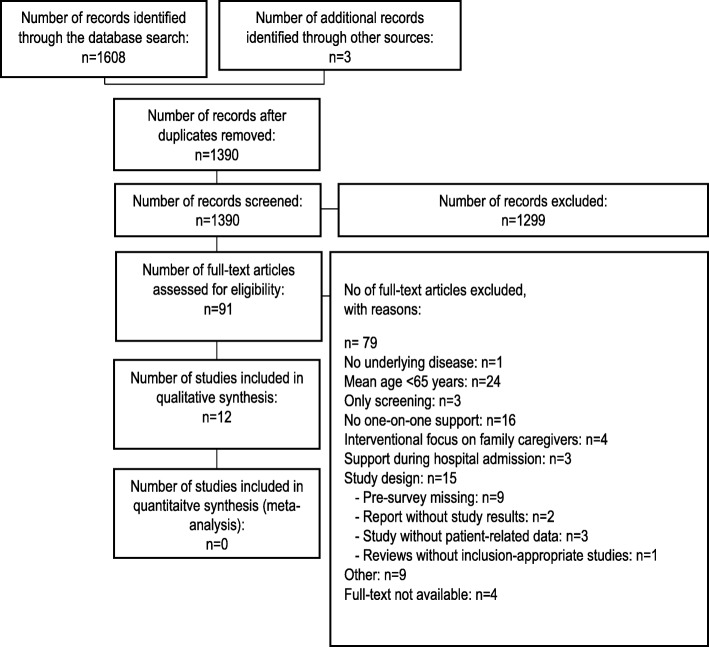
Flow diagram of the study-selection process (PRIMSA)

A total of 1608 records was identified, of which 218 duplicates were removed and 1299 records were excluded (Fig. 1). The independent screening of a random 20% sample of the remaining 91 full-text articles resulted in an agreement rate of 94.7%. Following full-text assessment for eligibility, a total of 12 studies were included in the analysis. The flow-diagram of the study-selection process is presented in Fig. 1. The full search and study-selection process is presented in Additional file 2. Due to the heterogeneous nature of the interventions and outcome measurements, a meta-analysis could not be conducted. Instead, the results are presented narratively. Due to the publication practice of the original authors, there was no primary publication available for some studies; as such, we explored the associated sub-publications. To fully reflect the different publications, all researched references are listed in this review. An overview of all cited publications is given in Tables 1–7.

**Table 1:** Study characteristics and populations

Study	Design	Country	Setting	Sample	Inclusion criteria (Age + Primary indication)	Female gender	Mean age (standard deviation)	Living alone
Psychosocial–coordinative support								
White et al. 2012 [111]	RCT	Australia	At home after hospital	649	18–80, Colorectal cancer	40.5%	64.9 (−)	–
Philippi et al. 2015 [108, 109, 112, 130]	CCT	Germany	At home after hospital	244	65+, need for social support (self-developed screening with cut-off value)	69.3%	76.3 (6.3)	70.1%
Physical–cognitive activation								
Haider et al. 2017 [113–116, 127]	RCT	Austria	At home after hospital	80	65+, Prefrail, frail, malnourished	83.8%	82.8(8.0)	75.0%
Etkin et al. 2006 [117]	PP	USA	At home	105	60+, Frail, homebound	86%	78.2(−)	–
Stolee et al. 2012 [119]	PP	Canada	At home	33	55+, Prefrail, frail	68.0%	80.0(8.8)	–
Connelly 2008 [118]	PP	Canada	At home	314	55+, Prefrail, frail, isolated	–	79.9(8.6)	55.7%
Assistance with medication intake								
Wang et al. 2013 [120]	RCT	Taiwan	At home after hospital	62	65+, At least two chronic diseases	54.8%	71.3(7.8)	35.5%
Sales 2013 [121]	RCT	USA	At home after hospital	137	18+, Congestive heart failure	57.7%	72.6(64.1)	–

*CCT* clinical controlled trial, *RCT* randomized controlled trial, *PP* studies with a single-group pre–post design

**Table 2:** Summary of interventions and data collection

Study	Intervention group	Length of intervention period	Ø Volunteer–patient contact time per month	Control group	Data collection			
					Baseline	During intervention	Postintervention	Follow-up(3 months)
Psychosocial–coordinative support								
White et al. 2012 [111]	M (P)	0.5–9 months	13–120 min	Care as usual	X	X	X	
Philippi et al. 2015 [108, 109, 112, 130]	V (H)	3 months	1161 min	Care as usual	X		X	X
Physical–cognitive activation								
Haider et al. 2017 [113–116, 127]	V (H)	3 months	516 min	Social support	X		X	
Etkin et al. 2006 [117]	V (H)	4 months	Not reported	No control group	X		X	
Stolee et al. 2012 [119]	V (H)	3 months	Not reported	No control group	X		X	
Connelly 2008 [118]	V (H)	3 months	Not reported	No control group	X		X	
Assistance with medication intake								
Wang et al. 2013 [120]	V (H + P)	2 months	218 min	Care as usual	X		X	
Sales 2013 [121]	M (P)	1 month	65 min	Care as usual	X		X	

*H* volunteer–patient contact by home visits; *M* Multiprofessional intervention, volunteers and professionals have contact with patients; *P* volunteer–patient contact by phone calls; *V* Patient contact solely by volunteers, professionals in background

**Table 3:** Outcomes and significant results

Outcome	Study	Outcome (original title)	Instrument^a^	Mean (standard deviation) / sample size (percent) at baseline		BL-Postintervention^b^			BL-Follow up (3 months)^c^		
						Diff.	d	p	Diff.	d	p
Psychosocial–coordinative support											
Anxiety	White et al. 2012 [111]	Anxiety	HADS (≥8)	IG	60(20%)	–	–	<.01			
				CG	53(16%)						
(Health-related) Quality of life	Philippi et al. 2015 [108, 109, 112, 130]	Social participation \(prevalence)	LLFDI-K	IG	3.1(0.7)				+ 0.2	.33	.002
				CG	3.3(0.6)						
Use of services	White et al. 2012 [111]	Use of services	Counting	IG	1.1(1.2)	-1,2	−1.0	<.001			
				CG	1.9(1.2)						
Further outcomes	White et al. 2012 [111]	Colorectal symptoms	CCSC	IG	3.0(2.0)	+ 0.3	.14	.02			
				CG	2.7(2.1)						
	Philippi et al. 2015 [108, 109, 112, 130]	Self-efficacy expectation	S-JS	IG	2.7(0.7)				+ 0.2	.33	.032
				CG	2.8(0.6)						
		Need for support	MOS-SS	IG	(−)71.8%	–	–	.022	–	–	.022
				CG	(−)47.7%						
Physical–cognitive activation											
Anxiety	Haider et al. 2017 [113–116, 127]	Fear of falling	FES-I	IG	44.1(13.07)	+ 3.9	.34	.016			
				CG	41.4(12.07)						
(Health related) Quality of life	Haider et al. 2017 [113–116, 127]	Activities	WHOQOL-OLD	IG	49.7(16.3)	–	–	.039			
				CG	53.8(14.3)						
	Etkin et al. 2006 [117]	Social functioning.	SF-20	IG	62.8(−)	+ 11.4	–	.003			
	Stolee et al. 2012 [119]	QOL (outside)	QOL-Scale	IG	69.6(29.2)	+ 7.9	.26	.001			
		QOL (at home)	QOL-Scale	IG	51.2(33.3)	+ 10.0	.30	.009			
Physical functioning	Haider et al. 2017 [113–116, 127]	Physical activity	PASE	IG	33.8(37.1)	+ 13.8	.44	<.001			
				CG	31.7(31.7)						
		Physical performance	SPPB	IG	5.2(2.9)	+ 0.9	.32	.044			
				CG	4.8(2.8)						
	Stolee et al. 2012 [119]	Balance confidence	ABC-Scale	IG	44.1(19.9)	+ 6.6	.33	.02			
		Chair stand	SFTM	IG	4.95(2.7)	+ 2.0	.74	<.001			
		Reaching forward	SFTM	IG	5.7(3.2)	+ 1.6	.05	.028			
Assistance with medication intake											
Use of services	Sales 2013 [121]	Readmission for heart failure (overall)	Counting	IG	–	–	12% ARR	.04			
				CG	–						
Further outcomes	Sales 2013 [121]	Composite endpoints (overall)	Counting	IG	–	–	25% ARR	.02			
	Wang et al. 2013 [120]	Knowledge	KAB-MS	IG	5.6(1.6)	+ 0.8	.28	.012			
				CG	4.8(2.9)						
		Behavior	KAB-MS								
		- when you get medication		IG	–	–	–	.013			
				CG	–						
		- Before you take medication		IG	–	–	–	.003			
				CG	–						
		- Caring for surplus medications		IG	–	–	–	.025			
				CG	–						

^a^ Further information on the instruments can be found in the original studies; ^b^ effect from the beginning of treatment until the end of treatment; ^c^ effect from the beginning of treatment until three months after the end of treatment; *ABC-Scale* Activities-Specific Balance Confidence Scale, *ARR* absolute risk reduction, *CCSC* Colorectal Cancer Symptoms Checklist, *CG* control group, *d* Effect size Cohen’s d, *Diff* Mean Difference, *FES-I* Falls Efficacy Scale – International, *HADS* Hospital Anxiety and Depression Scale, *IG* intervention group, *KAB-MS* Knowledge, Attitude, Behavior Medication Safety questionnaire, *LLFDI-K* Late Life Function and Disability Instrument – Short version, *MOS-SS* Medical Outcome Study Social Support Scale, *p* Significance p, *PASE* Physical Activity Scale for the Elderly, *QOL-Scale* Quality of life scale, *SF-20* Short-Form-Health Survey, 20 Items, *SFTM* Senior Fitness Test Manual, *S-JS* General self-efficacy, Jerusalem & Schwarzer (1986), *SPPB* Short Physical Performance Battery, *WHOQOL-OLD* World Health Organization Quality of Life for 60+

**Table 4:** Overview of significant and non-significant results

Outcome	Instrument*	Significant*	Non-significant*	Study
Psychosocial–coordinative support				
Anxiety	**HADS (≥8)**	**X**		[111]
	**HADS**		**X**	[108, 109, 112, 130]
Depression	**HADS (≥8)**		**X**	[111]
	**HADS**		**X**	[108, 109, 112, 130]
(Health-related) Quality of life	**LLFDI-K**	**X**	**X**	[108, 109, 112, 130]
	**SF 8, K − 14 F-SozU**		**X**	[108, 109, 112, 130]
Use of services	Counting	X		[111]
	Counting		X	[108, 109, 112, 130]
Further outcomes	CCSC	X		[111]
	**SCNS**		**X**	[111]
	**S-JS, MOS-SS**	**X**		[108, 109, 112, 130]
Physical–cognitive activation				
Anxiety	FES-I	X		[113–116, 127]
(Health-related) Quality of life	WHOQOL-BREF		X	[113–116, 127]
	WHOQOL-OLD	X	X	[113–116, 127]
	SF − 20	X	X	[117]
	ADL-Scale	X		[119]
	GAS	–	–	[118]
Physical functioning	PASE, SPPB	X		[113–116, 127]
	**SHARE-FI**, NMA®-LF		**X**	[113–116, 127]
	ABC-Scale	X		[119]
	SFTM	X	X	[119]
	BBS		X	[119]
	GAS	–	–	[118]
Assistance with medication intake				
Use of services	**Counting**	**X**		[121]
Further outcomes	**Counting**	**X**	**X**	[121]
	**KAB-MS**	**X**	**X**	[120]

* Bold fonds label primary outcomes and their results, ABC-Scale Activities-Specific Balance Confidence Scale, *ADL-Scale* Activities of daily living scale, *BBS* Berg Balance Scale, *CCSC* Colorectal Cancer Symptoms Checklist, *FES-I* Falls Efficacy Scale – International, *GAS* Goal Attainment Scaling, *HADS* Hospital Anxiety and Depression Scale, *K-14 F-SozU* Questionnaire to social support, *KAB-MS* Knowledge, Attitude, Behavior Medication Safety questionnaire, *LLFDI-K* Late Life Function and Disability Instrument, Short Version, Dimension ‘Impairment’, *MOS-SS* Medical Outcome Study Social Support Scale, *NMA®-LF* Mini Nutritional Assessment Long-Form, *PASE* Physical Activity Scale for the Elderly, *SF 8* Short-Form-Health-Survey – short, *SF-20* Short-Form-Health Survey – long, *SFTM* Senior Fitness Test Manual, *SHARE-FI* Assessment for frailty by Romero-Ortuno 2010 – handgrip strength, *S-JS* General Self-efficacy by Jerusalem & Schwarzer (1986), *SCNS* Supportive care needs survey, *SPPB* Short Physical Performance Battery, *WHOQOL-BREF* World Health Organization Quality of Life short version, *WHOQOL-OLD* World Health Organization Quality of Life for 60+

**Table 5:** Risk of bias (Cochrane, Acrobat-NRSI)

	Random sequence generation (selection bias)	Allocation concealment (selection bias)	Blinding of participants and personnel (performance bias)	Blinding of outcome assessment (detection bias)	Incomplete outcome data addressed (attrition bias)	Selective reporting (reporting bias)	Other sources of bias
Psychosocial–coordinative support							
White et al. 2012 [111]	+	–	–	–	–	?	+
Philippi et al. 2015 [108, 109, 112, 130]		-^1^	–	-^2^	–	+	+ ^3^
Physical–cognitive activation							
Haider et al. 2017 [113–116, 127]	+	+	-^s^	-^s^	+^s^	+	–
			+^o^	+^o^	+^o^		
Etkin et al. 2006 [117]						?	?
Stolee et al. 2012 [119]						?	?
Connelly 2008 [118]						?	?
Assistance with medication intake							
Wang et al. 2013 [120]	?	?	-^s^	?^s^	?	?	?
Sales 2013 [121]	?	?	-^s^	+^s^	?	?	?
			+^o^	+^o^			

+: low risk of bias;?: unclear risk of bias; −: high risk of bias

^1^Special type of Selection Bias for non-randomized Studies following Acrobat-NRSI; ^2^ Attrition Bias for non-randomized Studies following Acrobat-NRSI; ^3^ Bias in measurement of interventions (Acrobat NRSI); ^o^: objective Outcome; ^s^: subjective Outcome

**Table 6:** Summary of volunteer characteristics

Study	Sample size	Female gender	Mean age (standard deviation)	Prior specific knowledge
Psychosocial–coordinative support				
White et al. 2012 [111]	57	93%	–	Heterogeneous
Philippi et al. 2015 [108, 109, 112, 130]	35	85.7%	61.2(9.7)	Mainly no
Physical–cognitive activation				
Haider et al. 2017 [113–116, 127]	–	–	50+	Mainly no
Etkin et al. 2006 [117]	103	89%	53.2(−)	–
Stolee et al. 2012 [119]	59	–	–	Heterogeneous
Connelly 2008 [118]	113	90%	50.9(20.7)	–
Assistance with medication intake				
Wang et al. 2013 [120]	11	55%	45.3(6.6)	Yes
Sales 2013 [121]	6	–	–	Yes

**Table 7:** Overview of training concepts

	Duration (h)	Number of appointments	Participant limit	Theoretical background	Checklist completed	Contents of the training							Didactic methods								Evaluation methods		Further support		
						Get to know each other	Frame and structures	Sensitization of target group	Tasks (theory)	Tasks (practice)	Self-care	Summary and reflection	(Group) discussion	Partner interviews	Lecture	Group work	Case scenarios	Role playing / practice	Video	Written information	Feedback	Test	Group meetings	Telephone hotline	Manual/handouts
Psychosocial–coordinative support																									
White et al. 2012 [111]	18	3	15	E	Yes	✓	✓	✓	✓	✓	✓	✓	✓	–	✓	✓	✓	✓	✓	✓	–	✓	✓	–	✓
Philippi et al. 2015 [108, 109, 112, 130]	16	3	15	C	Yes	✓	✓	✓	✓	✓	✓	✓	✓	✓	✓	✓	✓	✓	–	✓	✓	–	✓	✓	✓
PEQ [93, 128, 129]	30	10	8–15	SDL	–	✓	✓	✓	✓	✓	✓	✓	✓	✓	✓	✓	✓	✓	✓	✓	✓	–			✓
Physical–cognitive activation																									
Haider et al. 2017 [113–116, 127]	12	4	25	(C)	Yes	–	✓	✓	✓	✓	✓	–	–	–	✓	✓	–	✓	–	✓	✓	–	✓	✓	✓
Etkin et al. 2006 [117]	16	2	–	EOSD	No								✓		✓		✓	✓	✓	✓	✓		✓		✓
Stolee et al. 2012 [119]	12	2	–	–	No																✓				
Connelly 2008 [118]	–	–	–	–	No																✓				
Assistance with medication intake																									
Wang et al. 2013 [120]	26	6	6	–	No			✓	✓													✓	✓		
Sales 2013 [121]	–	–	11	–	No																				✓

*C* Constructivism, *E* Experimental, *EOSD* Elements of successful dissemination, *SDL* Self-determined learning (constructivism)

The twelve included studies were published between 2003 and 2017; five were RCTs, two were CCTs, and five were PPs. The total number of participants was 3379; one study had less than 50 participants, four studies had 50–99 participants, five studies had 100–500 participants, and two studies had more than 500 participants. Five studies were conducted in the USA, two in Canada, and one each in Australia, Austria, Germany, Finland, and Taiwan, respectively. The mean age of the study participants was 77.0 years (range: 67.3–82.8; *n* = 10), 66.2% of the participants were female (range: 40.0–90.0%; *n* = 11), and 60.6% lived alone (range: 32.8–100%; *n* = 8). The last point of data collection from the 10 studies followed postintervention, while for one study it occurred during the intervention [107] and three months postintervention [108, 109], respectively.

To meet the heterogeneity of the different interventions within studies, we categorized them on the basis of the tasks the volunteers performed. In the categorization process, it became clear that the data were not sufficient to form well-supported categories. Therefore, we used categories derived from a scoping review, which summarized 77 non-pharmacological treatments of dementia in geriatric mental health institutions [110]. We added to these initial categories by inductively identifying additional types of volunteer support: coordinative, domestic, and medication-intake support.

- **Psychosocial support** focuses on the psyche and social well-being – e.g., the strengthening of emotional well-being and social inclusion.
- **Coordinative support** focuses on strengthening organizational and coordinative skills – e.g., the support in the contact and use of a nursing service.
- **Physical activation** focuses on physical condition and fitness – e.g., strength and skills are trained by manualized exercises.
- **Cognitive activation** focuses on cognition – e.g., orientation in space and time or training of short- or long-term memory.
- **Domestic support** focuses on help in the household – e.g., help with shopping, cooking, or cleaning.
- **Assistance with medication intake** focuses on the correct intake of medication – e.g., by taking a medication plan or by providing home visits.

The interventions could not be clearly assigned to one of the categories, as they usually combined components of different categories. After examining the interventions for overlap and various combinations, we identified six “fields of activity”; three of these were implemented as treatments after a hospital stay and are presented in detail hereinafter.

### Fields of activity evaluated as treatment after hospital (presented in detail)

- Within the realm of **psychosocial–coordinative support**, the volunteers assumed emotional, psychosocial, and organizational/coordinative support. An RCT [111] and a CCT [108, 109, 112] evaluated this type of support following a hospital stay.
- Within the realm of **physical–cognitive activation**, the volunteers assumed manualized exercises in a patient’s home. An RCT [113–116] and three PPs [117–119] evaluated this type of support. The RCT followed a hospital stay [113–116], while the PPs took place in a domestic setting [117–119].
- Within the realm of **assistance with medication intake**, the volunteers reminded elderly of their medication intake after a hospital stay. Two RCTs [120, 121] evaluated this type of support following a hospital stay.

### Categories that were initially coded, but are not presented in the review as they did not occur after a hospital stay

- Within the realm of **psychosocial–domestic support**, volunteers assumed emotional, psychosocial, and home help to the elderly in domestic settings. One CCT (*n* = 1520) evaluated this type of support [122].
- Within the realm of **physical–cognitive activation plus social participation (outdoors)**, the volunteers assumed manualized exercises and joint outdoor activities in solely domestic settings. One RCT (*n* = 121) [123, 124] and one PP (*n* = 64) [125, 126] evaluated this type of support.

Given that the sixth field of activity, **coordinative support**, did not appear to be voluntary in nature, it was excluded post hoc. The details of this intervention were comparable with a statutory regulated engagement in Germany referred to as legal support. With this type of intervention, volunteers assumed organizational and coordinative support to the elderly after a hospital stay. The target group was comprised of persons who lacked an appropriate decision maker or private guardianship. One retrospective PP study (*n* = 50) evaluated this type of support [107].

To obtain information on volunteer training we:

1. Obtained checklist information on volunteer training from three studies: two concerning psychosocial–coordinative support [108, 109, 111, 112] and one concerning physical–cognitive activation [113–116, 127].
2. Analyzed the published training information from the remaining five studies [117–121].
3. Identified one further curriculum through an institutional search that reported on 32 training concepts; this fell under psychosocial–coordinative support and was called “PEQ” [93, 128, 129].

The study characteristics and populations are summarized in Table 1; for specific volunteer tasks and intervention details, see Table 2; for the results, see Table 3 and Table 4; for a summary of risk of bias, see Table 5.

### Psychosocial–coordinative support

*Definition, data basis, and target group:* Within psychosocial–coordinative support, the volunteers assumed emotional, psychosocial, organizational, and coordinative tasks. An RCT [111] and a CCT [108, 109, 112] identified this type of support. The support addressed persons with health restrictions (e.g., those with colorectal cancer) who had a simultaneous need for support. Philippi et al. [108, 109, 112] included elderly individuals who were not yet in need of care, but who needed social support as they had received insufficient support from the immediate social environment. The authors excluded persons with insufficient cognitive capacity, psychiatric disorders, and those with care provided through a nursing care insurance fund. Study characteristics and populations are summarized in Table 1.

*Specific tasks of the volunteers*: The intervention designed by White et al. [111] was based on four telephone calls, each lasting approximately 30 min. During the intervention period, the volunteers offered emotional, informational, and instrumental support; each support type offered was needs-oriented, and in the event that ambiguity arose, support was offered in consultation with professionals. In Philippi et al. [108, 109, 112], the volunteers offered weekly home visits, each lasting 2–4 h. The support included organizational support (e.g., accompanying patients to a doctor’s visit, providing bureaucratic support, and aiding in the search for professional support), psychosocial support (e.g., emotional/social support through conversation), and leisure activities (e.g., walks or sports). For intervention details, see Table 2.

*Results*: The primary outcomes of the studies were anxiety [108, 109, 111, 112], depression [108, 109, 111, 112], quality of life [108, 109, 112], unmet needs [111], general self-efficacy [108, 109, 112], and social support [108, 109, 112]. Single studies showed small to medium positive effects on anxiety [111], social participation [108, 109, 112], colorectal symptoms [111], self-efficacy expectations [108, 109, 112], and the need for support [108, 109, 112]. The positive effect on anxiety demonstrated by White et al. [111] could not be confirmed in Philippi et al.’s study [108, 109, 112]. Neither study had an observable effect on depression. In both studies, the control group showed less service use; in fact, White et al. [111] demonstrated that this effect was highly significant with a large effect size. None of the studies reported adverse events. For additional details, see Table 3 and Table 4.

*Study quality*: Both studies had a high risk of bias in terms of allocation concealment, blinding of participants and personnel, blinding of outcome assessments, and incomplete outcome data [108, 109, 111, 112] (see Table 5). No intention-to-treat (ITT) analyses were conducted. White et al. lost power by falling below the calculated sample size by about 3.1% [111], while Philippi et al. fell by about 22.3%, mainly due to the small intervention group (*n* = 47) [108, 109, 112]. The participant retention at last observation was 82.1% for White et al. [111] and 67.2% for Philippi et al. [108, 109, 112]. Thus, the reported effects might be overestimated [106]. For a summary, see Table 5.

### Physical–cognitive activation

*Definition, data basis, and target group:* Within physical–cognitive activation, the volunteers assumed manualized training in the patient’s home. An RCT [113–116] and three PPs [117–119] evaluated this type of support. All activations were tailored toward prefrail and frail elderly [113–119]. Haider et al. excluded persons with insufficient cognitive capacity (MMSE ≤17) and several conditions that might serve as a contraindication to receiving physical training (e.g., chemo- or radiotherapy, insulin-treated diabetes, or chronic obstructive pulmonary disease III or IV) [113–116], while the PP studies lacked specification of their exclusion criteria. For study characteristics and populations, see Table 1.

*Specific tasks of the volunteers:* The intervention designed by Haider et al. [113–116] consisted of two home visits per week, each lasting about one hour. During the intervention period, the volunteers offered standardized activation, comprising three components: (1) physical training (warm up, six standardized strength exercises with an elastic band, and repetitions, individualized as required), (2) a nutritional program (a discussion of one nutritional issue at each home visit), and (3) social support (conversation or cognitive training). In Etkin et al. [117], the volunteers offered weekly home visits. The activation included a videotape with warm-up exercises, which included 11 strengthening exercises using thera-bands, a cool-down, and cognitive behavioral strategies (e.g., a motivational video). Two PPs [118, 119] offered weekly physical training. The training included 10 [118] and 15 [119] standardized exercises, respectively. For three studies, the participants were supposed to train independently [117–119]. For intervention details, see Table 2.

*Results:* The primary outcome of the RCT was frailty, specifically handgrip strength [127]. The RCT showed medium positive effects on fear of falling, quality of life (activities), physical activity [115], and physical performance, but not on handgrip strength [113]. The positive effect on (health-related) quality of life was confirmed in Etkin et al. [117] and Stolee et al. [119]. Further, Stolee et al. [119] found positive effects on physical functioning. (Health-related) quality of life and physical functioning had both significant and non-significant results within the single studies. Two studies reported on adverse events: Haider et al. reported on one participant with back pain (1.25%) and four participants (5%) who could not be re-examined due to death or a medical decision (not caused by the intervention) [113–116]. Etkin et al. [117] reported that there were no serious adverse events, but participants also reported injuries or pain (*n* = 16; 15.2%; e.g., joint, muscle, or limb pain, and shortness of breath). For additional details, see Table 3 and Table 4.

*Study quality:* Within the RCT, there was a risk of “other sources of bias” due to the fact that the volunteers offered both the intervention and the control intervention (social support) [113–116]. Additionally, data that were scheduled to be obtained at the 6- and 12-month follow-up sessions had not yet been published [127]. Participant retention at the last observation was 83.8%; as such, for the post-intervention data, an ITT analysis was conducted [113–116]. With regard to the presented post-intervention data, the risk of overestimated results appeared to be low [106]. Participant retention in the PP studies amounted to 53.5% [117], 53.1% [119], and 79.3% [118]. For a summary, see Table 5.

### Assistance with medication intake

*Definition, data basis, and target group:* Within this type of intervention, the volunteers reminded the participants of their medication intake. Two RCTs [120, 121] evaluated this type of support. The support was intended for individuals with at least two chronic diseases [120] or congestive heart failure [121]. Both studies excluded patients with dementia. Sales et al. additionally excluded patients with severe psychiatric disorders [120, 121]. For study characteristics and populations, see Table 1.

*Specific tasks of the volunteers:* The intervention designed by Wang et al. [120] was based on three home visits (each lasting about 2 h) and three phone calls (each of about 0.5 h in duration). During the first home visit, the volunteer coached the participant using a manual and reminder sticker. During second and third home visits, participants were reminded of their prescriptions and safety behaviors. Support for prescription adherence was also offered through phone calls. In Sales et al. [121], the volunteers offered participants an in-hospital visit at discharge day, followed by weekly, manualized telephone calls (each lasting about 15 min). At the hospital, the volunteers reviewed the patients’ medications and advised each patient to schedule appointments with their cardiologist. Within 24–48 h after discharge, the first phone call took place, and the patient’s progress and results were shared with the cardiologist. For intervention details, see Table 2.

*Results:* The primary outcome of the studies included readmission for heart failure, worsening heart failure [121], and medication safety knowledge, attitudes, and behaviors [120]. The studies showed small to medium positive effects on readmission for heart failure [121], medication safety knowledge, and several components of medication safety behavior [120]. No effects were found on all-cause mortality [121] and medication safety attitudes [120]. None of the studies reported adverse events. For details, see Table 3 and Table 4.

*Study quality:* Risk of bias could not be appropriately assessed due to a lack of data. For a summary, see Table 5.

### Who are the volunteers?

The volunteers who offered psychosocial–coordinative support were mostly female and, on average, about 60 years old. They primarily had no or varied prior specific knowledge. The volunteers who offered physical–cognitive activation were about 10 years younger, and they had to be in sufficient physical condition to engage with patients. When examining assistance with medication intake, the volunteers were again slightly younger, comprising volunteers with previous experience as hospital volunteers [120] and premedical students [121]. For a summary, see Table 6.

### Training for volunteers

All training took place before the first volunteer–patient contact. The training lasted between 13 and 26 h, with a mean appointment duration of between three and six hours. The mean group size was 15 (range: 6–25). The training contained the sections “get to know each other”, “frame and structures”, “sensitization to the target group”, “tasks (theory)”, “tasks (practice)”, “self-care”, and “summary and reflection”, whereby the different interventions detailed varying priorities. The various training sessions were primarily carried out by study staff and occasionally supplemented by external trainers (e.g., legal experts [108, 109, 112]). The trainers were qualified in the fields of medicine, psychology, social sciences, and nursing [108, 109, 112–115, 120, 127]. Training on physical–cognitive activation also involved dieticians, physical therapists, and sports scientists [113–115, 117, 121, 127]. A summary of the training concepts, along with information on training contents, didactic methods, evaluation methods, and further support, are presented in Table 7.

Within “tasks (theory/practice)”, psychosocial–coordinative support focused on conversational skills [93, 111, 112, 128], while physical–cognitive activation and assistance with medication intake focused on repetitive exercise practice [113, 127] and knowledge on medication safety [120], respectively. As lectures were primarily used for “frame and structures” and “tasks (theory)”, activating techniques (e.g., discussions, partner interviews, group work, case scenarios, and role playing) were commonly used for “get to know each other”, “sensitization to the target group”, “tasks (practice)”, and “self-care”.

The training concepts were evaluated via post-training feedback [93, 108, 109, 112–119, 127–129]; occasionally, volunteers were tested on theoretical concepts or practical applications [111, 120]. The results of these evaluations have rarely been published. Overall, the findings that have been published indicated a high level of satisfaction with the training [108, 117–119]. During the intervention, the volunteers received support in one to three monthly group meetings [108, 111, 113, 117, 120], telephone hotlines [108, 113, 117], and manuals/handouts [108, 111, 113, 117, 121].

## Discussion

The review identified three evaluated fields of one-on-one-interventions for the multimorbid elderly, which were offered by trained volunteers and implemented following discharge from hospital: Psychosocial–coordinative support, physical–cognitive activation, and assistance with medication intake.

The number and quality of identified trials remains limited; specifically, the long-term effects of these interventions have not yet been investigated or published. The results indicate that psychosocial-coordinative support may have had short-term effects on anxiety, quality of life, and additional outcomes in persons who require this type of support [108, 109, 111, 112]. Moreover, there is evidence indicating that physical–cognitive activation has an impact on anxiety and physical functioning in persons with frailty [113–119], and assistance with medication intake has an effect on service use and medication safety in persons with complex medication profiles [120, 121]. Verification of these effects on the primary outcomes have partially failed [109, 111, 113], and proven effects within and between studies remain inconsistent [109, 111, 116, 117, 119–121]. The proven short-term effects are mostly small or medium sized; based on the currently available data, a meta-analysis could not be conducted, partly because patients with numerous clinical indications were excluded from these studies. As such, there is a lack of knowledge in this field, especially when investigating supports for persons with dementia.

Based on these results, further international studies should be performed to verify the effectiveness of the different one-on-one supports offered by volunteers to the multimorbid elderly following discharge from hospital. Countries such as Germany or Sweden – where the volunteer sector is already established and comprised of many dedicated people – can play a pioneering role in this regard [15].

Based on our investigation, the mean age of volunteers ranged from 45 to 61 years; the age gap between patients and trained volunteers was between 29.0 years [118] and 15.1 years [109]. In the field of psychosocial–coordinative support, elderly volunteers seemed to support elderly patients (mean age: 61.2 years vs. 76.3 years) [109]. Accordingly, this approach may be one appropriate way to address the impact of demographic changes on the healthcare system. An analysis from Germany confirmed this: In the voluntary field of “care”, there are a disproportionate number of older, female, well-qualified, already retired persons [11]. In future studies, these age-related characteristics should be investigated internationally.

This review was conducted in conjunction with the study “Local, collaborative, stepped and personalized care management for older people with chronic diseases – a randomized comparative effectiveness trial” (German Clinical Trial Register, ID: DRKS00013904). One component of the study is the use of trained volunteers as providers for one-on-one support for chronically ill, multimorbid elderly after a hospital stay. The results of this review contributed to the development of the training. The training curricula for volunteers were rarely published, and the details of these programs could only be obtained by contacting the authors of the various studies investigated herein. In view of the claim that volunteers should be well prepared for their service, but that they may not replace professionals [14], it is important to understand the background and effects of volunteer training. Specifically, current publication practices do not consider the special nature of volunteer deployment.

This review now provides a systematic overview of training and one-on-one interventions, as well as their associated effects. The limited number of studies included, as well as incomplete training backgrounds (despite the use of checklist surveys) did not enable direct conclusions to be drawn between training and intervention outcomes. But based on our findings, we can draw some conclusions about volunteer training programs: First, group sizes and training duration depend on the type of intervention in question. While psychosocial–coordinative support and assistance with medication intake had smaller group sizes (10–15 participants), physical–cognitive activation had bigger groups (up to 25 participants). This could be due to the rationale that focusing on psychosocial conversation techniques or complex medications requires closer accompaniment than providing training on standardized physical exercises. Second, the total duration of trainings on psychosocial–coordinative support and assistance with medication intake was of longer duration (range: 16–30 h) than the total duration of trainings on physical–cognitive activation (range: 12–16 h). The longer training period resulted from extensive units on “Sensitization of the target group” and “Tasks (theory / practice)”.

Third, due to the mean age of the volunteers, it generally seems useful to leverage the tenets of adult education. Looking at theoretical influences within the studies, one trend becomes visible: Three training concepts adopted constructivist learning theory to justify their curriculum (Philippi et al. [108, 109, 112, 130], partially Haider et al. [113–116], and the curriculum “PEQ” [128]) Those three came from German-speaking authors. Based on the available information on the other studies, no other trends on the theoretical background of their trainings could be identified.

Constructivist concepts are characterized by the perception that adults are “capable of learning”, but that they cannot be “instructed” [131]. Subsequently, knowledge instruction was replaced by enabling active processes of construction of knowledge; in response, claims of heteronomy were declined, while self-determined and self-organized, situated concepts of learning emerged [132–135]. Since the 1990s, German-speaking concepts of adult education have been increasingly affected by constructivist approaches [136]. Several studies showed the efficacy of constructivist approaches in adult education, particularly on the transfer of knowledge in practice [137, 138]. In Germany, several approved training programs for volunteers that support the elderly already exist; they have demonstrated effectiveness among patients and include constructivist participation procedures that the herein presented concepts lacked [89, 91]. An international discussion on the (different) appropriate theoretical backgrounds of volunteer training with implications for future study designs is needed.

Since the trainings were primarily carried out by study staff, study-based funding is assumed. Similarly, volunteering also involves permanent costs [14], such as those associated with the provision of volunteers’ qualifications, accompaniment, and insurance. If the interventions are to find their way into care practice in the long term, sustainable financing models will be needed.

### Strengths and limitations

Our systematic review has several strengths: We conducted an extensive electronic and institutional search (see Additional file 2). The supplementary institutional search generated additional results. It would be worthwhile repeating the institutional search with an international working group. To adequately comply with the different intervention durations, we considered the duration of follow-up from the end of the intervention. To handle the heterogeneity of the interventions, we developed a brief typology of the various interventions that were implemented following discharge from hospital; to compensate for the lack of published data, we examined the details of volunteer training by checklist. Both provided implications for future practice and research. By assessing primary and secondary outcomes, we presented a comprehensive review of an insufficiently studied field of research.

The review also has some limitations: We waived registration of the review protocol due to the procedural approach of the review. Registration was not feasible before the full-text articles were identified and extracted. To comply with the heterogeneity of interventions and assessment instruments, we did not perform a meta-analysis. The fact that we limited our search period to 15 years may have influenced the results. Previous systematic reviews on associated issues had a broader search period; however, they included only a few studies published before 2002 [94, 139]. A longer search period would probably not have led to major changes in the results, but it may have influenced the results, as we excluded formalized volunteer services from our research. It is likely that larger providers of formalized volunteer services evaluated their training programs, even if they did not publish them. However, it could not be assumed that pre–post evaluations without study-based funding were widespread in the nonprofit-sector, and the formalized volunteer services that we had excluded addressed volunteers who were willing to commit to a fixed commitment period with a high number of working hours. Including these types of volunteers and their related training programs could have also biased the conclusions. Further, as we focused the institutional search on Germany, our results may have been influenced. However, none of the cited studies were found during the institutional search; that being said, an international search would likely not have led to major changes in the main results. However, it should be noted that the theoretical trend “constructivist learning” is also based on the PEQ curriculum [128], which was found through the institutional search. Therefore, an international institutional search could have made further theoretical training trends visible. That we had to extend the inclusion criterion to include “at least one chronic primary diagnosis” due to current tagging practice in the databases may have also influenced our results. However, due to the high proportion of multimorbid conditions among those over 65 years of age [103], a high proportion of multimorbid patients could be expected in the identified studies. A certain risk of bias may have emerged since the screening of records and data extraction were performed by a single author. Independent screening of 20% of random records by a second author resulted in substantial agreement.

## Conclusions

New approaches are needed to address the challenges associated with the demographic changes, staff shortages, and societal changes associated with providing care for the elderly. The main implication of the review findings is that psychosocial–coordinative support, physical–cognitive activation, and assistance with medication intake may be effective volunteer-based interventions in the one-on-one support of multimorbid, chronically ill elderly at the interface between hospital and domesticity (offered by a non-formalized volunteer service). However, there are only a few studies on this topic and the results are inconsistent. Therefore, the hypothetical effects of the different types of voluntary support require prespecified logic models. Thus, the impact of volunteering in this area can be better described with improved data and enhanced impact levels. Further studies should be oriented toward the identified fields, particularly as they relate to continuing treatment following discharge from hospital. These interventions are designed for specific patient groups and feature defined support principles, precise inclusion criteria, and accurate volunteer training. Due to the volunteers’ age, it seems that psychosocial–cognitive activation is most suitable for coping with the current demographic change. The suitability and transferability of the different fields of continuous treatment following discharge from hospital on country-specific settings should be discussed and examined in feasibility studies. Furthermore, an international discussion on the (different) appropriate theoretical backgrounds of volunteer training with implications for future study designs is needed. Implemented training concepts urgently need to be evaluated and published, ideally following the principles of the TIDieR checklist and guideline [100].

## Additional files

Additional file 1: PRISMA Checklist. Full checklist of PRISMA reporting guidelines. (PDF 621 kb)

Additional file 2: Search strategy for Medline (PubMed). Example of the search strategy. (PDF 603 kb)

## Abbreviations

ABC-Scale: Activities-Specific Balance Confidence Scale
Acrobat NRSI: A Cochrane risk of bias assessment tool: for non-randomized studies of interventions
ADL-Scale: Activities of daily living scale
ARR: Absolute risk reduction
BAGFW: Federal Association of Non-statutory Welfare, Germany
BAGSO: German National Association of Senior Citizens’ Organisations
BBE: National Network for Civil Society, Germany
BBS: Berg Balance Scale
BMBF: Federal Ministry of Education and Research
BMFSFJ: Federal Ministry for Family Affairs, Senior Citizens, Women and Youth, Germany
BMG: Federal Ministry of Health, Germany
C: Constructivism
CCSC: Colorectal Cancer Symptoms Checklist
CCT: Controlled clinical trial
CG: Control group
d: Effect size Cohen’s d
Diff.: Mean Difference
E: Experimental
EOSD: Elements of successful dissemination
ESS: European Social Survey
FES-I: Falls Efficacy Scale - International
GAS: Goal Attainment Scaling
GKV: National Association of Statutory Health Insurance Funds, Germany
H: Volunteer-patient contact by home visits
HADS: Hospital anxiety and depression scale
IG: Intervention Group
ITT: Intention-to-treat analysis
K − 14 F-SozU: Questionnaire to social support
KAB-MS: Knowledge, attitude, behavior medication safety questionnaire
LLFDI-K: Late Life Function and Disability Instrument – Short Version
M: Multiprofessional Intervention, volunteers and professionals have contact with patients
MeSH: Medical Subject Headings
MOS-SS: Medical Outcome Study Social Support Scale
NMA®-LF: Mini-Nutritional Assessment Long Form
p: Significance p
P: Volunteer-patient contact by phone-calls
PASE: Physical Activity Scale for the Elderly
PEQ: Curriculum “Pflege, Engagement und Qualifizierung”
PI(C)OS model: Search strategy including participants, intervention, control group, outcomes and study design
PP: Single-group pre-post design
PRISMA: Preferred Reporting Items for Systematic Reviews and Meta-Analyses
QOL-Scale: Quality of life scale
RCT: Randomized controlled trial
SCNS: Supportive care needs survey
SDL: Self-determined learning (constructivism)
SF-20: Short-Form-Health Survey, 20 Items
SF-8: Short-Form-Health Survey, 8 Items
SFTM: Senior Fitness Test Manual
SHARE-FI: Assessment for frailty by Romero-Ortuno 2010 – handgrip strength
S-JS: General self-efficacy, Jerusalem & Schwarzer, 1986
SPPB: Short Physical Performance Battery
TIDieR: Template for Intervention Description and Replication
V: Patient contact solely by volunteers, professionals in background
VdK: Social Welfare Association, Germany
WHOQOL-BREF: World Health Organization Quality of Life, short version
WHOQOL-OLD: World Health Organization Quality of Life for 60+
