# Supplementary material for: Trained volunteers to support chronically ill, multimorbid elderly between hospital and domesticity – a systematic review of one-on-one-intervention types, effects, and underlying training concepts
Source: BMC Geriatr. 2019 May 2;19:126. doi: 10.1186/s12877-019-1130-2 (PMC6498473; doi:10.1186/s12877-019-1130-2)
Supplement: Supplementary file 2 — Search strategy for Medline (PubMed). Example of the search strategy. (PDF 603 kb) [file 12877_2019_1130_MOESM2_ESM.pdf]

# Additional file 2

| Search strategy for Medline (PubMed) | |
|---|---|
| #1 | (Aged[Title/Abstract]) |
| #2 | (Aged[MeSH]) |
| #3 | (aged, 80 and over[Title/Abstract]) |
| #4 | (aged, 80 and over[MeSH]) |
| #5 | (elderly[Title/Abstract]) |
| #6 | (geriatric patients[Title/Abstract]) |
| #7 | (multiple chronic conditions[MeSH]) |
| #8 | (multiple chronic conditions[Title/Abstract]) |
| #9 | (chronic disease[MeSH]) |
| #10 | (chronic disease[Title/Abstract]) |
| #11 | (chronic[Title/Abstract]) |
| #12 | (ongoing condition [Title/Abstract]) |
| #13 | (persistent illness*[Title/Abstract]) |
| #14 | (persistent disease*[Title/Abstract]) |
| #15 | (long term illness*[Title/Abstract]) |
| #16 | (long term disease*[Title/Abstract]) |
| #17 | (ongoing health condition [Title/Abstract]) |
| #18 | (health impairments[Title/Abstract]) |
| #19 | (degenerative [Title/Abstract]) |
| #20 | (degenerative disease[Title/Abstract]) |
| #21 | (neurodegenerative [Title/Abstract]) |
| #22 | (neurodegenerative diseases [Title/Abstract]) |
| #23 | (neurodegenerative diseases [MeSH]) |
| #24 | (Comorbidity[MeSH]) |
| #25 | (Comorbidity[Title/Abstract]) |
| #26 | (comorbid[Title/Abstract]) |

| | |
|---|---|
| #27 | (Multimorbid[Title/Abstract]) |
| #28 | (multimorbidity[Title/Abstract]) |
| #29 | (dementia[MeSH]) |
| #30 | (dementia[Title/Abstract]) |
| #31 | (alzheimer disease[Title/Abstract]) |
| #32 | (alzheimer disease[MeSH]) |
| #33 | (diabetes mellitus[Title/Abstract]) |
| #34 | (diabetes mellitus[MeSH]) |
| #35 | (diabetes mellitus, type 2[MeSH]) |
| #36 | (diabetes mellitus type 2[Title/Abstract]) |
| #37 | (Diabetes[Title/Abstract]) |
| #38 | (depressive disorder[Title/Abstract]) |
| #39 | (depressive disorder[MeSH]) |
| #40 | (depressive disorder, major[MeSH]) |
| #41 | (major depression[Title/Abstract]) |
| #42 | (frail[Title/Abstract]) |
| #43 | (frailty[Title/Abstract]) |
| #44 | (ambulatory care[MeSH]) |
| #45 | (ambulatory care[Title/Abstract]) |
| #46 | (domestic[Title/Abstract]) |
| #47 | (community[Title/Abstract]) |
| #48 | (community service[Title/Abstract]) |
| #49 | (home care setting*[Title/Abstract]) |
| #50 | (homecare setting*[Title/Abstract]) |
| #51 | (home-based[Title/Abstract]) |
| #52 | (home rehabilitation[Title/Abstract]) |
| #53 | (home health aides[Title/Abstract]) |
| #54 | (home health care[Title/Abstract]) |
| #55 | (home care services [Title/Abstract]) |

| | |
|---|---|
| #56 | (home care services[MeSH] |
| #57 | (outpatients[Title/Abstract]) |
| #58 | (outpatients[MeSH]) |
| #59 | (outpatient service[Title/Abstract]) |
| #60 | (hospitals[MeSH]) |
| #61 | (hospitals[Title/Abstract]) |
| #62 | (Clinic[Title/Abstract]) |
| #63 | (Emergency service, hospital[MeSH]) |
| #64 | (Emergency service, psychiatric[MeSH]) |
| #65 | (emergency care[Title/Abstract]) |
| #66 | (patient discharge[MeSH]) |
| #67 | (patient discharge[Title/Abstract]) |
| #68 | (hospital discharge[Title/Abstract]) |
| #69 | (Aftercare[Title/Abstract]) |
| #70 | (Aftercare[MeSH]) |
| #71 | (elder care [Title/Abstract]) |
| #72 | (health services for the aged[MeSH]) |
| #73 | (health services for the aged[Title/Abstract]) |
| #74 | (volunteers[MeSH]) |
| #75 | (hospital volunteers[MeSH]) |
| #76 | (hospital volunteers[Title/Abstract]) |
| #77 | (lay help*[Title/Abstract]) |
| #78 | (family companion*[Title/Abstract]) |
| #79 | (companion model*[Title/Abstract]) |
| #80 | (care companion*[Title/Abstract]) |
| #81 | (Lay led[Title/Abstract]) |
| #82 | (Lay leader*[Title/Abstract]) |
| #83 | (Lay expert*[Title/Abstract]) |
| #84 | (Lay worker*[Title/Abstract]) |

| | |
|---|---|
| #85 | (lay health worker*[Title/Abstract]) |
| #86 | (Lay person*[Title/Abstract]) |
| #87 | (Lay advisor*[Title/Abstract]) |
| #88 | (lay health advisor*[Title/Abstract]) |
| #89 | (Lay educator*[Title/Abstract]) |
| #90 | (Lay health educator*[Title/Abstract]) |
| #91 | (Lay tutor*[Title/Abstract]) |
| #92 | (Lay instructor*[Title/Abstract]) |
| #93 | (Layperson[Title/Abstract]) |
| #94 | (voluntary-worker*[Title/Abstract]) |
| #95 | (volunteer worker*[Title/Abstract]) |
| #96 | (Trained volunteer*[Title/Abstract]) |
| #97 | (volunteer* aide*[Title/Abstract]) |
| #98 | (Voluntary work*[Title/Abstract]) |
| #99 | (Voluntary care*[Title/Abstract]) |
| #100 | (Voluntary service*[Title/Abstract]) |
| #101 | (Voluntary support*[Title/Abstract]) |
| #102 | (Voluntary involvement[Title/Abstract]) |
| #103 | (Voluntary help*[Title/Abstract]) |
| #104 | (Voluntary counsel*[Title/Abstract]) |
| #105 | (Voluntary staff[Title/Abstract]) |
| #106 | (Voluntary provide*[Title/Abstract]) |
| #107 | (Voluntary program*[Title/Abstract]) |
| #108 | (Voluntary sector*[Title/Abstract]) |
| #109 | (Voluntary health agencies[MeSH]) |
| #110 | (Voluntary health agencies[Title/Abstract]) |
| #111 | OR/ #1-#43 |
| #112 | OR/ #44-#73 |
| #113 | OR/ #74-#110 |

| | |
|---|---|
| #114 | AND/ #111-#113 |
| #115 | #114, Limiter Publication Date: 2002/01/01-2017-04-01 |
| #116 | #115, Limiter Language: English, German |
| #117 | #116, Limiter Aged: 65+years |
